# The Jekyll and Hyde story of IL17-Producing γδT Cells

**DOI:** 10.3389/fimmu.2015.00037

**Published:** 2015-02-04

**Authors:** Rushikesh S. Patil, Sajad A. Bhat, Asif A. Dar, Shubhada V. Chiplunkar

**Affiliations:** ^1^Chiplunkar Laboratory, Advanced Centre for Treatment, Research and Education in Cancer (ACTREC), Tata Memorial Centre, Kharghar, India

**Keywords:** γδT cell, IL17, Tγδ17, infection, inflammation, cancer

## Abstract

In comparison to conventional αβT cells, γδT cells are considered as specialized T cells based on their contributions in regulating immune response. γδT cells sense early environmental signals and initiate local immune-surveillance. The development of functional subtypes of γδT cells takes place in the thymus but they also exhibit plasticity in response to the activating signals and cytokines encountered in the extrathymic region. Thymic development of Tγδ1 requires strong TCR, CD27, and Skint-1 signals. However, differentiation of IL17-producing γδT cells (Tγδ17) is independent of Skint-1 or CD27 but requires notch signaling along with IL6 and TGFβ cytokines in the presence of weak TCR signal. In response to cytokines like IL23, IL6, and IL1β, Tγδ17 outshine Th17 cells for early activation and IL17 secretion. Despite expressing similar repertoire of lineage transcriptional factors, cytokines, and chemokine receptors, Tγδ17 cells differ from Th17 in spatial and temporal fashion. There are compelling reasons to consider significant role of Tγδ17 cells in regulating inflammation and thereby disease outcome. Tγδ17 cells regulate mobilization of innate immune cells and induce keratinocytes to secrete anti-microbial peptides thus exhibiting protective functions in anti-microbial immunity. In contrast, dysregulated Tγδ17 cells inhibit Treg cells, exacerbate autoimmunity, and are also known to support carcinogenesis by enhancing angiogenesis. The mechanism associated with this dual behavior of Tγδ17 is not clear. To exploit, Tγδ17 cells for beneficial use requires comprehensive analysis of their biology. Here, we summarize the current understanding on the characteristics, development, and functions of Tγδ17 cells in various pathological scenarios.

## Introduction

Decades have passed since the accidental discovery of T cells expressing γ and δ chains (1), yet it is hard to define γδT cells like αβT cells. Ambiguity in understanding the functions of γδT cells is attributed to their unparalleled characteristics as compared to αβT cells. Current understanding of T cell biology has emerged extensively from studies on αβT cells; however, recent findings have underlined the crucial role of γδT cells in shaping the immune response in infections, inflammatory diseases, and cancer. They are involved in early immune response like innate cells, produce proinflammatory cytokines (IFNγ, IL17, and TNFα), and activate adaptive immune cells. The cytokines secreted by γδT cells determine their effector functions. In humans, the major cytokine produced by γδT cells is IFNγ, contributing to its role in anti-viral, anti-bacterial, and anti-tumor immunity (2–4). However, upon activation γδT cells can be skewed toward IL17, IL4, or TGFβ producing phenotype governed by the polarizing cytokines present in the surrounding milieu (5). Recent investigations in mice and human have highlighted the role of IL17-producing γδT cells (hereafter referred as Tγδ17) in bacterial infection, inflammatory disease, and cancer (6–8). They are the primary source of IL17 in early disease condition and are pivotal in progression and disease outcome (9, 10). To understand the functional significance of Tγδ17 in pathological conditions, many efforts have made in mouse models but there is scanty literature available on human Tγδ17 cells. In this review, we will discuss the recent findings of Tγδ17 differentiation, mechanisms regulating IL17 production, and their relevance in pathological conditions.

## γδT Cells: Unique but Versatile

Survival of γδT cells over strong evolutionary selection pressure highlights their exclusive importance and disparate properties from conventional αβT cells. Initially, γδT cells were considered as cells of innate immunity owing to their ability to recognize conserved non-peptide antigens expressed by stressed cells. In addition to this, they recognize pathogen-associated molecular pattern (PAMP) or danger-associated molecular pattern (DAMP) through pattern recognition receptors (PRR) expressed by them (11). Like adaptive immune cells, human γδT cells undergo clonal expansion and exhibit antigen-specific memory (12). Thus, γδT cells link innate and adaptive immunity thereby enhancing the immune response against invading pathogen or danger signal posed by “self” cells. Antigen recognition by murine or human γδT cells does not require antigen presentation by major histocompatibility complex (MHC) class I or class II (13) and the crystal structure of γδTCR has revealed its close homology with immunoglobulins suggesting that antigen recognition by γδT cells is similar to antigen–antibody interaction (14). However, diversity of antigens recognized by γδT cells brands it different from B cells. The antigens exclusively recognized by γδT cells are not peptides of protein antigens rather are small mono- and pyrophosphates of linear C5 isoprenoids called as phosphoantigens (13). These prenyl pyrophospahtes are metabolites of cholesterol biosynthesis and are recognized through complementarity determining regions (CDRs) of γδT cells (15). In humans, during cholesterol biosynthesis, phosphorylated precursors such as isopentenyl pyrophosphate (IPP) and DMAPP (dimethylallyl pyrophosphate) are synthesized by mevalonate pathway (16). However, microbial pathogens use non-mevalonate pathway to produce these phosphorylated precursors (17). γδT cells respond to these natural or synthetic stimulators with varying degree. Based on this, stimulators are classified either as weak or potent stimulators. HMBPP [(E)-4-hydroxy-3-methyl-but-2-enyl pyrophosphate], a metabolite of non-mevalonate pathway of bacteria *Mycobacterium tuberculosis* is 10^4^ times more potent stimulator of human γδT cells than IPP (18). The exclusive response of γδT cells to these phosphoantigens has a potential therapeutic significance and synthetic pyrophosphates can be used to harness the cytotoxic potential of γδT cells.

Murine and human γδT cells also recognize phycoerythrin (PE) – fluorescent molecule of cyanobacteria and red algae. PE is directly recognized by γδT cells but there is no sequence similarity between PE-specific murine and human γδ TCR (19). Naturally occurring primary alkyl amines activate human Vγ2Vδ2 T cells and enhance immunity against certain microbes and plant-derived antigens (20, 21). Similar to natural killer (NK) cells, human γδT cells also recognize the stress-induced MHC class I-related molecules MICA, MICB, and the UL16-binding proteins that are upregulated on malignant or stressed cells (22, 23). The stress-related molecules are ligands for NKG2D expressed by γδT cells and this engagement also enhances γδT cells’ response to non-peptide antigens (24). Human and murine γδT cells recognize lipid antigens presented by CD1 molecules, a classical ligand for NK T cell suggesting the phenomenon similar to MHC-restricted antigen recognition by αβT cells (25–27). The murine γδT cells also recognize non-classical MHC class I molecules like T10 and T22 (β2 microglobulin-associated molecules lacking peptide binding groove) (28, 29). In addition to non-protein and MHC related antigens, murine and human γδT cells also recognize small peptides such as heat shock proteins (HSPs) (30–32). However, they do not require antigen-presenting cells (APCs) and recognition of antigen is MHC unrestricted, resembling B cells (33). Thus, the broad spectrum antigen responsiveness of γδT cells helps them to mount faster immune response.

Like αβT cells, γδT cells develop in the thymus from CD4^−^CD8^−^ (double negative, DN) thymocytes (34); however, they precede αβT cells in T cells ontogeny. γδ TCR rearrangements can be traced in early embryonic stages in mice as well as in humans (35, 36). This highlights their role in neonatal protection as conventional T cells are functionally impaired and APCs are immature in newborns (37). During thymic development, the decision of γδ versus αβ T cell commitment is determined by TCR signal strength or notch signaling (38). In mice, the strong TCR signaling in absence of notch signal induces γδT cells lineage commitment whereas low TCR signal strength in presence of strong notch signaling promotes αβ T cell lineage (39–41). However, notch signaling alone is insufficient to decide γδ/αβ T cell commitment. The intrinsic signals from T cell receptor complex and trans-conditioning by different subsets of thymocytes also determine thymic development of γδT cells (42). In humans, notch has opposite role in αβ versus γδT cell lineage decision, sustained notch signaling is required for the development of γδT cells (43) which is determined by differential notch receptor–ligand interaction importantly Jagged2/Notch3 signaling (44). In human, γδT cells differentiate along two pathways, a notch-independent DN pathway, generating mature DN and CD8αα^+^ SP (single positive) TCRγδ^+^ cells. In the notch-dependent DP (double positive) pathway, immature CD4^+^ SP, and subsequently DP TCRγδ^+^ cells are generated. Human postnatal thymus thus exhibits a scenario of DN, DP, and SP TCRγδ^+^ population, which highlights heterogeneity in human γδT cell development (45). The activated extrathymic γδT cells, in humans, express notch receptors, which regulate their effector functions. Inhibiting notch signaling in γδT cells dampened their anti-tumor cytotoxic potential (46). Thus, validates the requirement of notch signaling in both thymic development and functions of human γδT cells. The diversity of human γδ T cell repertoire at birth (majorly contributed by Vδ1^+^ subset of γδT cells in cord blood) is restricted in adulthood especially to Vγ9Vδ2, a circulating subset of γδT cells. The absolute numbers of Vγ9Vδ2 T cells increase from minor population at birth to more than 75% of γδT cells pool in peripheral blood (35), which constitute around 1–10% of total T cells in humans. The γδT cells exit the thymus as mature T cells and express markers that are associated with antigen-experienced T cells (47).

The other important feature of γδT cells apart from antigen recognition is their tissue tropism. In humans, the first γδT cells to arise from thymus are Vδ1^+^ (paired with various Vγ chains), which preferentially populate in epithelial tissue and constitute larger proportion of intraepithelial lymphocytes (IELs) (48). They rapidly and innately recognize stressed cells found to be enriched in various tumor tissues (4). The Vγ9Vδ2 is a lymphoid homing subset of γδT cells, which continually expand in response to microbial antigen in circulation and exhibit characteristics of adaptive immune system (49). These cells recognize, expand, and secrete cytokines in response to non-peptide antigens associated with microbes in circulation. In mouse, a substantial proportion of γδT cells reside as the IEL in the skin, intestine, and genitourinary tract. In response to the chemokine signals, Vγ5Vδ1^+^ T cells leave the fetal thymus, reside in the epidermis, and form dendritic-like network similar to Langerhans cells. These cells are called as dendritic epidermal T cells (DETCs) and constitute more than 90% of epidermal T cells (50). Vγ6^+^ T cells home to tongue and reproductive tract whereas Vγ7^+^ T cells home to intestinal tract suggesting that distinct TCR repertoire are present at different anatomical site and respond to antigens unique to their resident tissues (51–53). However, the functions of IELs are determined by the environment at the anatomical site (54) and hence specific γδ T cell subset could be used in tissue repair and generation of effective immune response at different epithelial sites.

γδT cells perform diverse effector functions determined by the TCR expressed, tissue localization, and activation status. Apart from these, MHC-independent recognition of antigens, production of IFNγ, and expression of cytotoxic granules classify γδT cells as potential cytotoxic cells (55). They can kill activated, infected, stressed, and transformed cells using various strategies such as engagement of death-inducing receptors, such as FAS and TNF-related apoptosis-inducing ligand receptors (TRAILR) and the release of cytotoxic effector molecules such as perforin and granzyme (56, 57). Human γδT cells also recognize HSP (HSP60/70) expressed on tumor cells and enhance its cytolytic activity against the tumors (31, 58). γδT cells support the maturation and activation of other lymphocytes, NK cells, and macrophages with the help of secreted chemokines (CCL3, CCL4, CXCL10) (55). Another chemokine CXC–chemokine ligand 13 (CXCL13) produced by Vγ9Vδ2 cells can regulate B cell organization within lymphoid tissues and help B cells to produce antibodies (59). Human γδT cells can also crosstalk with dendritic cells (DCs) influencing each other functions like the antigen presentation by DCs, activation, and secretion of IL12 and IFNγ by γδT cells, which result in DC maturation (11, 60). These properties of γδT cells aid in generation of the effective immune response in the appropriate condition. Not only this, activated Vγ9Vδ2 cells can take up and process the soluble antigens, opsonize target cells, and can migrate to lymph nodes through CC-chemokine receptor 7 (CCR7) where they upregulate expression of MHCs and co-stimulatory receptors CD80 and CD86 (61, 62). Activated Vγ9Vδ2 cells has also been licensed to act as APC and activate CD4 and CD8 T cells (63). Collectively, these observations highlight the multi-talented role of γδT cells, having both Th- and Tc-like properties along with acting as APC. The special trait of γδT cells is their ability to recognize phosphorylated non-protein antigens and mediate its effector function in spatial and temporal manner making them a robust cell type, which can be manipulated to develop a promising tool for novel immunotherapies against certain types of diseases. However, care should be adapted while designing such immunotherapies because these cells have capacity to secrete various cytokines under different conditions.

## Tγδ17: A Subtype of γδ T Cells

Unlike αβ T cells, in mice, which leave thymus as naïve cells and are primed in the peripheral compartment, γδT cells undergo subset commitment in the thymus itself. However, in humans, upon activation with different cytokines, Vγ9Vδ2 cells can be polarized toward different effector subtypes like γδ1, γδ2 (64), γδ17 (65, 66), and γδTreg (67, 68). This functional plasticity of γδT cells assists them to tackle the distinct disease conditions and play important role in the early responses to invasive pathogens. The recent findings have stated that γδT cells are major IL17 producers and have shown their involvement in early onset of immune activation (69). Similar to Th17 cells, Tγδ17 cell express RORγt as a lineage determination transcriptional factor (70). Healthy adult human peripheral blood Vγ9Vδ2 T cells distinctively express Th1 signature and 50–80% produce IFNγ but <5% produce IL17 (6). However, Tγδ17cells have been demonstrated to be involved in the pathogenesis of transplantation rejection (71), autoimmune disease (72), allergy (73), and cancer (74) in humans. The biology of Tγδ17 is so naive that it compels us to cross-examine its genesis, functions, and clinical relevance to understand its therapeutic potential.

## Molecular Evidences of Tγδ17 Genesis

The molecular mechanism of IL17-producing γδT cells remains an enigma. Most of the studies carried out to understand the differentiation mechanisms of Tγδ17cells are based on the murine models. γδT cells preferentially localized to barrier tissues are the initial source of IL17 and are likely to originate from the fetal thymus. These are called as the natural IL17-secreting γδT cells. γδT cells that make IL17 within 24 h fall in this category (75). γδT cells acquire IL17-secreting phenotype in secondary lymphoid tissues after antigen exposure, which is referred to as induced Tγδ17 cells (76, 77).

**Figure 1:**
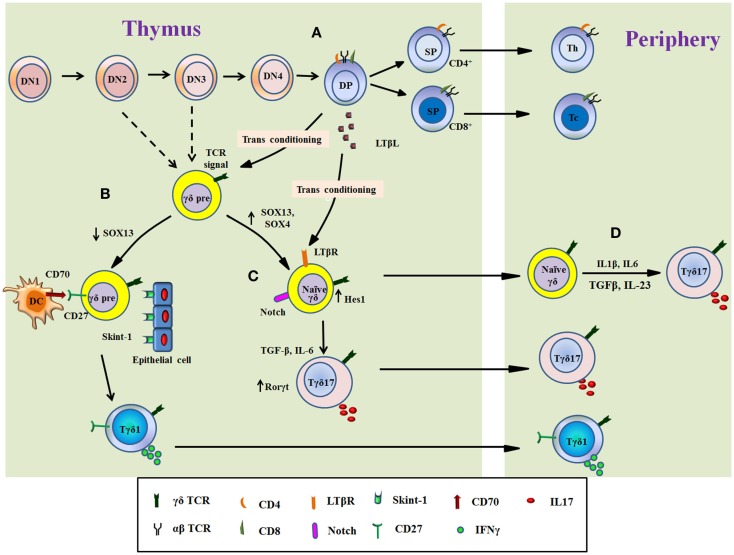
**Overview of Tγδ17 cells development**. The figure illustrates the differentiation of Tγδ17 cells from T cell progenitors in the murine thymus **(A–C)** and from naïve γδT cells in periphery in human **(D)**. Progenitor T cells differentiate through double negative stage 1 (DN1) to DN stage 4 **(A)**. The decision of αβ or γδ TCR expression takes place at early T cells precursor (from DN2 or DN3 stage) as showed by dashed line. The thymocytes expressing αβ TCR develop into double-positive thymocytes, which support differentiation of functional subtypes of γδT cells called as transconditioning. DP thymocytes secrete LTβL, which support differentiation of Tγδ17. The DP αβ thymocytes then exit the thymus as mature single positive T cells (either CD4^+^ or CD8^+^ T cells) **(A)**. The functional programing of γδT cells is determined by TCR signal and/or other related signals. TCR signal, interaction with Skint-1 from epithelial cells, downregulation of SOX13, and signaling through CD27/CD70 divert γδ thymocytes toward IFNγ-producing phenotype (Tγδ1), which migrate to periphery **(B)**. Conversely, signaling through Notch receptor maintain Sox13 levels with increase in Hes1 and RORγt expression induce γδ thymocytes to produce IL17. Progression of γδ thymocytes to Tγδ17 cells is independent of signaling through Skint-1 and/or CD27 but require inputs from IL6 and TGFβ. The natural Tγδ17 cells developed in thymus migrate to tissue or periphery **(C)**. In human, naïve γδT cells, which exit thymus, can also differentiate into Tγδ17 cells in presence of TCR signal and cytokines such as IL6, IL1β, IL23, and TGFβ **(D)**.

During development of T cells in thymus, murine γδT cells branch off at the transition of thymocytes from DN3 stage to DN4 stage (34). It is also reported that γδT cells develop from DN2 stage and specifically produce IL17 whereas IFNγ-producing γδT cells can develop from both DN2 and DN3 precursors (78) (Figure 1). This suggests that γδT cells do not develop like αβT cells and follow evolutionary ancient path of T cell development. However, the precise DN stage from which γδT cells develop is elusive (79). Fetal thymic γδ T-cell development occurs in successive waves by using the different Vγ and Vδ segments during the embryonic development (34, 80). Successful gene rearrangement of γδ T cells from early thymic precursors (CD44^hi^) lead to the development of naïve γδ T cell characterized by CD44^lo^ CD27^+^CD62L^+^ phenotype. This phenotype can either leave the thymus to populate in secondary lymphoid organs or it can undergo further intrathymic differentiation that results in the development of multiple γδ T cell subtypes such as dendritic epidermal γδT cell (DETCs), Tγδ17, or NK 1.1^+^ γδ cell (γδNKT cells) (80, 81). Recently, it was described that when thymic lobes of mice at E14 were colonized with DN1a cells from mice at E13 and E18, respectively. It was observed that although both populations (E13 DN1a cells and E18 DN1a cells) generated similar number of γδT cells, only E13 DN1a cells generated Vγ3^+^ DETCs. These observations indicate that precursor lineage of DETCs may be different and needs further investigation (82). DETCs develop at embryonic day 13 (E13) to approximately E17 and readily secrete IFNγ when activated. After the development of DETCs, the next functional developmental wave consists of Tγδ17 cells. Tγδ17 cells are heterogeneous in using TCR chains that mainly include Vγ6^+^ and Vγ4^+^ but also use Vγ1^+^ chain. Vγ6^+^ cells develop by E14 to around birth and finally Vγ1 and Vγ4 cells develop E16 onward (81). The other subtypes of γδ T cells, which develop in thymus, are γδ NKT cells, which are similar to invariant TCRαβ^+^ NKT cells (83, 84).

There are different thymic signaling processes, which determine functional phenotype of γδT cells in thymus before migration to periphery and contribute to the balance between IFNγ committed versus IL17-commited subtypes (85). This biasness toward IL17 or IFNγ depends on the antigen experience in thymus. The γδ T cells that have encountered the cognate antigen interaction in thymus, gain the potential to differentiate into the IFNγ-producing functional phenotype while antigen naïve γδ T cells develop into IL17-producing γδT cells (86). This skewedness also reflects in their distribution outside the thymus. Most of Tγδ17 cells reside in lymph nodes whereas IFNγ-producing γδT cells are mainly found in the spleen and the mechanism for this distribution is not clear (86). Similar distribution is also found in αβT cells and it seems to be logical as the lymph nodes serve as the site of initial exposure to foreign antigens and propagate the wave of inflammation, thus are suited for the earliest source of the IL17 secretion (87).

Besides the γδ TCR signaling (86), expression of tumor necrosis factor receptor family member, CD27, determines the IL17 versus IFNγ production by γδT cells (88). CD27^+^ γδT cells differentiate into IFNγ producing cells whereas IL17 production was restricted to CD27^−^ T cells (89) (Figure 1). Thus thymic “imprinting” of the γδT cells as CD27^+^ or CD27^−^ regulates effector functions of γδT cells and is preserved in the periphery (89). CD27 is not only associated with IFNγ production but also aids γδT cells to interact with its ligand CD70 expressed on DCs, thymic epithelial cells, and double-positive thymocytes thus acting as a costimulatory receptor (89). Therefore, CD27 conveys an intrathymic message that licenses the CD27^+^ γδ T cells for the production of IFNγ (47). Another signaling pathway that influences the differentiation of Tγδ17 is the signaling through lymphotoxin-β receptor (LTβR), a member of the tumor necrosis factor receptor family (90). Signaling through LTβR leads to the activation of the alternative nuclear factor (NF)-κB pathway via RelB. Ligands for LTβR regulating this developmental process are produced by CD4^+^CD8^+^ thymocytes (91). The homeostasis of this functional phenotypic differentiation, influenced by other thymic progenitors is known as transconditioning (91), which highlights coordination between different signaling pathways in thymus that occur in physically separate thymic niche (92). LTβR signaling pathway controls Tγδ17 development by regulating transcription factors RORγt and RORα4, required for IL17 expression in γδ thymocytes (93). The role of LTβR signaling, however, remains controversial as LTβR is present downstream to CD27 signaling, which is associated with the IFN-γ production (89).

The maturation of Tγδ17 cells from its precursors requires TCR signaling as mice with reduced ZAP70 show decreased number of Tγδ17 cells (94). However, TCR signaling alone is not sufficient as it also requires other signals (95). An src family kinase, Blk (B lymphoid kinase), is required for Tγδ17 cells development in thymus as Blk-deficient mice was reported to have less number of IL17-producing γδ T cells (96). Similarly, high-mobility group (HMG) box transcription factors, SOX4 and SOX13 are positive regulators of Tγδ17 development (95, 97). These transcription factors expressed in immature T cells (98) highlight that the development of Tγδ17 is from early precursors (DN2) (78, 95). Other thymic determinant, which is responsible for the functional dichotomy in Tγδ17 and Tγδ1, is Skint-1, a thymic epithelial cell determinant. The interaction between Skint-1^+^ cells and γδ thymocytes (Vγ5^+^Vδ1^+^) induce an Egr3-mediated pathway, leading to differentiation toward IFNγ-producing γδ T cells. Further, it suppresses Sox13 and an RORγt transcription factor-associated Tγδ17 cells lineage differentiation suggesting that the functions of the earliest T cells are substantially preprogramed in the thymus (99). Notch signaling is known to be involved in thymic determination and development of Tγδ17 cells. Hes1, one of the basic helix–loop–helix (bHLH) proteins induced by Notch signaling is critical for the IL17 expression by γδ T cells and its thymic development (100–102). Further, the specific expression of Hes1 in CD25^+^ and CD27^−^ γδ T cells and decreased levels of Tγδ17 in Hes1-deficient mice highlights the critical role of Notch–Hes1 pathway in Tγδ17 development in thymus as well as in periphery (101). The thymic development of Tγδ17 is independent of STAT3 but partly dependent on RORγt (101) and most peripheral IL17-producing γδ cells express RORγt and respond rapidly to IL23 (103).

Developmental process of Tγδ17 also requires signaling through different cytokines. TGFβ signaling is necessary for Tγδ17 development (104). It has been shown that in absence of TGFβ1 or Smad3 (a component of the TGFβ signaling), the number of Tγδ17 thymocytes reduced drastically relative to that of wild-type mice (104). As compared to TGFβ, requirement of IL6 for Tγδ17 development is not well understood as there are contrasting reports on its role (72, 105). It is also reported that IL6 does not act directly on uncommitted γδ thymocytes but instead it acts indirectly by regulating the expression of Delta-like ligand 4, a ligand for notch receptor, expressed by thymic epithelial cells that promote the differentiation of Tγδ17 (101, 106). Moreover, IL23 and IL1 produced by DCs are crucial for IL17 production by γδT cells. IL23^−/−^ and IL23R^−/−^ mice showed the significant reduction in Tγδ17 cells after *L. monocytogenes* infection supporting earlier observation (107–110).

Thymic development of human Tγδ17 cells is poorly investigated. Around 80% circulating human Vγ9Vδ2 T cells are IFN-γ producers and express CD27 whereas CD27 negative cells are IL17-producing γδ T cells are <5% (65). Interaction of CD70 with CD27 promotes the expansion of Th1-biased Vγ9Vδ2 T cells in periphery (111). However, such role in their thymic development is unknown. Human Vγ9Vδ2 T cells can be polarized to Tγδ17 cells in periphery upon IPP activation and in the presence of cytokines like TGFβ, IL1β, IL6, and IL23, followed by a week of culture in differentiation medium supplemented with IL2 can induce IL17 in these cells (65, 66). In humans, there are contrasting reports on role of IL6 and IL23 in differentiation of Tγδ17. It has been shown that IL6 is required for differentiation of neonatal Tγδ17, and IL23 is required for the generation of adult IL17-producing γδT cells (65). In another study, it is reported that in the presence of TCR signaling, IL23 promotes the induction of IL17 in neonatal (but not adult) γδT cells (112). However, it appears that IL23 induces γδT cells to coproduce IL17 and IFNγ in adults but support development of Tγδ17 cells in neonates. In addition to the above-mentioned cytokines, IL7 selectively promotes the mouse and human IL17-producing γδT cells. IL7 activates STAT3 preferentially in γδT cells competent to produce IL17 (113). However, the increased IL17 production by γδT cells upon TCR stimulation in presence of IL7 is observed only in case of cord blood cells but not with peripheral lymphocytes. Thus, it is important to note that the antigen naïve γδT cells only can be reprogramed *in vitro* toward Tγδ17 phenotype (66, 113).

The kinetic study of IL17 production by γδT cells has shown that murine γδT cells secrete IL17 within few hours after stimulation (70). This phenomenon can be reasoned by the thymic development of murine Tγδ17 cells and constitutive presence of transcriptional regulators for IL17 production. However, human γδT cells in thymus are functionally immature and can attain their functional differentiation in periphery in presence of cytokines (114). This supports the kinetics of IL17 production by human γδT cells that mRNA expression of IL17 and RORγt peaks by day 3–6 and decrease by day 9 onward, after stimulation. The expression of cytokine receptors (IL1βR, IL6R, TGFβR, and IL23R) on Vγ9Vδ2 T cells peaks on day 3 and decrease by day 6 (66). Thus, coordinated combination of TCR and cytokine stimulation could be necessary for the sustained secretion of IL17 by γδT cells, which highlights the difference in kinetics of IL17 secretion by murine and human Tγδ17 cells. This underscores that human γδ T cells can be “reprogramed” in the periphery into different functional lineages.

Upon antigenic challenge, T cells differentiate to memory phenotype; either central memory (TCM) or effector memory (TEM) (115). Human Tγδ17 cells present in non-lymphoid environment belong to CD27^−^ CD45RA^±^ effector (74) or terminally differentiated (TEMRA) (66) memory phenotype. Similarly, murine Tγδ17 cells also show effector memory phenotype with CD44^high^, CD45RB^low^, and CD62L^low^ (116). Thus, Tγδ17 cells differentiated either in thymus or in periphery, belong to memory phenotype, and licensed to patrol the blood, lymphoid organs, and peripheral tissues.

## Tγδ17 in Microbial Infections

Tγδ17 cells can rapidly produce IL17 upon Toll-like receptors (TLR) or cytokine stimulation alone even in absence of antigen presentation. The general proinflammatory functions of IL17 [reviewed in Ref. (117, 118)] could be associated with γδT cells as they are major producers of IL17. Studies carried out in various infection models showed that Tγδ17 cells are protective against infection. During mycobacterial infection, IL17 produced by Vγ4^+^ and Vγ6^+^ cells induce pulmonary granuloma formation by recruitment of granulocytes and monocytes. The IL17 participates in maturation of granuloma by promoting tight cell to cell binding via ICAM1 and LFA1 induction (119). Mycobacteria-infected DCs secrete IL23, which regulate IL17 production by γδT cells emphasizing that the early activation of Tγδ17 cells is important for initiating inflammation and recruiting innate immune cells to the site of infection thereby enhancing bacterial clearance from host (120, 121). Tγδ17 cells also support cell-mediated immunity by inducing Th1cells against pulmonary mycobacterial infection (122).

In *Escherichia coli* infection model also, γδT cells were reported to be the major producers of IL17, which enhanced neutrophil infiltration to the peritoneum. The infiltration of cells diminished after antibody depletion of resident Vδ1^+^ subtype of γδT cells highlighting its involvement in IL17 secretion in response to IL23 (9). Thus, IL23 and Tγδ17 cells play a dominant role as first line of defense in infection before CD4 T cell activation. In case of *L. monocytogenes* infection, a large number of γδ T cells accumulate in the lymph organs shortly after infection and begin to produce IL17A, signifying the role of Tγδ17 cells in the *Listeria* infection (123). IL17 was also shown to promote proliferation of CD8^+^ cytotoxic T lymphocytes by enhancing DC cross-presentation *in vitro*. DCs stimulated with IL17 showed upregulation of MHC-I molecule H2Kb and enhanced secretion of cytokines (IL12, IL6, and IL1β). CD8α^+^ DCs from *Il17a*^−/−^ mice also produced less IL12 and are less potent in activating naive CD8^+^ T cells (123). This indicate that Tγδ17 cells not only induce innate response but also critical for optimal adaptive cytotoxic response against intracellular bacterial infection. The alliance of IL23 and Tγδ17 is also demonstrated to have a protective role during infections such as *Klebsiella pneumonia* (124), *Citrobacter rodentium* (125, 126), *Salmonella enterica* (127, 128), and *Toxoplasma gondii* (129). The Tγδ17 cells also play a vital role in clearing fungal infections. The rapid production of IL17A was reported in the lungs at a very early stage after intravenous infection with *C. albicans*. Lung resident γδ T cells were the major source of early IL17A production regulated by IL23 and TLR2/MyD88-dependent pathway (130). Presence of Tγδ17 cells were also reported in the lungs of neutropenic mice during *C. neoformans* infection. These Tγδ17 cells played an important role in the chemotaxis of leukocytes and induction of protective immune response (131). Tγδ17 cells thus orchestrate the protective immunity by acting at the early onset in infection models (108).

Relatively few studies have evaluated the role of Tγδ17 cells in human microbial immunity. In patients with tuberculosis (TB), elevated levels of Tγδ17 cells were found in peripheral blood and were major producers of IL17 (6). As a protective role, in response to bacterial antigens, IL17-producing Vγ9Vδ2 T cells induce neutrophil migration through secretion of CXCL8 and promote their phagocytic activity (66). Tγδ17 cells also induce epithelial cells to secrete anti-microbial peptides like β-defensins in response to bacterial antigens (66). This signifies the modulatory effects of Tγδ17 cells on keratinocytes and other immune cells in anti-microbial defense. In children with bacterial meningitis, the population of IL17^+^ Vγ9Vδ2 T cells significantly increase in peripheral blood and at the site of infection (cerebrospinal fluid). The reversal of this pattern after successful anti-bacterial therapy clearly suggests the anti-microbial role of Tγδ17 cells (66). Collectively, these studies provide new insight into the functions of γδ T cells as the first line of host defense against bacterial and fungal infection in human and may pave a path in designing newer treatment modalities.

## Toll-Like Receptors Regulate IL17 Production in Tγδ17 Cells

γδT cells express various chemokine receptors, cytokine receptors, and PRRs, which regulate IL17 production. TLRs are the well-studied PRRs expressed by DCs, macrophages, and γδT cells. The unique microbial molecules called as PAMP are recognized by TLRs, which orchestrate the anti-microbial response in γδT cells (11). In malarial infection, MyD88 deficiency results in severe impairment of IL17A producing γδT cells levels, but not IFNγ producing γδT cells highlighting differential control by innate signaling through TLRs in infections (132). Murine Tγδ17 cells specifically express TLR1 and TLR2 but not TLR4. High number of Tγδ17 cells were induced upon *in vivo* stimulation with Pam3CSK4 (ligand for TLR2) but not with LPS (TLR4 ligand) or CpG (TLR9 ligand) (70). Interestingly, it has been shown that TLR4 indirectly controls IL17 generation by γδT cells through IL23 secreted by TLR4 expressing macrophages in response to HMG Box 1 (HMGB1, a damage-associated protein and TLR4 ligand) (133). Moreover, Tγδ17 cells promote experimental intraocular neovascularization (134) as well as early acute allograft rejection (135) in response to HMGB1. Signaling through TLR2 is indispensable for Tγδ17 in anti-microbial functions. Absence of TLR2 or MyD88 in cutaneous *Staphylococcus aureus* infection, or in *Candida albicans* infection, caused an impaired IL17 production and poor microbial clearance in the skin infiltrated with Vγ5^+^ γδT cells (130, 136). Tγδ17 cells also express DC-associated C-type lectin 1 (dectin 1) and intraperitoneal injection of curdlan (dictin 1 ligand), induced IL17 production by γδT cells (70). In imiquimod (IMQ)-induced psoriasis-like model, dermal γδT cells spontaneously secreted a large amount of IL17 in IMQ-treated skin cells. Thus, it appears that TLR7/8 (receptor of IMQ) may regulate the IL17 production by γδT cells. It is important to note that the modulatory effects of TLRs on γδT cells as showed in *in vivo* murine models are mediated through IL23 and/or IL1β cytokines. The direct stimulation of CD27^−^ γδT cells by TLR ligands (LPS or PAM) show no effect on IL17 production (132). This suggests that TLR signaling indirectly modulates Tγδ17 function.

## Receptor Repertoire Expressed by Tγδ17 Cells

The receptor profile of Tγδ17 cells is similar to Th17 cells. In mice, the majority of IL17-producing CD4 cells belong to CCR6^+^ compartment compared to CCR6^−^ (137). Sorted CCR6^+^ γδT cells showed increased mRNA expression of IL17, IL22, IL23R, Rorγt, and aryl hydrocarbon receptor (AhR) compared to CCR6^−^ γδT cells (70, 138). This suggests that CCR6 can be a phenotypic surface marker of Tγδ17 cells. Besides CCR6, Tγδ17 cells express various chemokine receptors including CCR1, CCR2, CCR4, CCR5, CCR7, CCR9, CXCR1, CXCR3, CXCR4, CXCR5, and CXCR6 (7). The early onset recruitment of Tγδ17 to the site of inflammation is determined by the type of chemokine receptor on them. Tγδ17 cells expressing CCR6 and CCR9 show selective migration toward allergic inflamed tissue in response to CCL25 (ligand for CCR9). α4β7 integrin expression is indispensable for this migration and transendothelial crossing of Tγδ17 cells. (139). Since migration through CCL2/CCR2 axis is determinant for total γδT cells, CCL25/CCR9-mediated migration seems to be specific for Tγδ17 subtype (140, 141).

In humans, Tγδ17 cells express CCR6 but not CXCR3, CXCR5, CCR3, CCR4, or CCR5. However, they express granzyme B, FASL, and TRAIL but not perforin (66). The lack of granzyme B and perforin coexpression may be responsible for absence of cytolytic activity of Tγδ17 cells. On the contrary, it has been shown that the human colorectal tumor-infiltrating Tγδ17 cells do not express FASL or TRAIL but express CD161 and CCR6 (74). The inconsistency in expression of cytolytic markers and their relevance on Tγδ17 cells needs to be understood in detail. The AhR is indispensable for Tγδ17 cells as it promotes differentiation of naïve Vγ9Vδ2 T cells toward Tγδ17 phenotype (66).

In mouse model, it has been shown that Ahr^−/−^ Tγδ17 cells express IL17 but fail to produce IL22 (70). Moreover, in mouse model of *Bacillus subtilis* induced pneumonitis, deficiency of Ahr resulted into low IL22 production but IL17 levels were maintained (142). Thus, although Ahr promotes IL17, it is indispensable for IL22 production by Tγδ17 cells.

## Inflammatory Disorders and Mania of Tγδ17

Th17 cells and Tγδ17cells are essential in disease progression and are pathogenic in autoimmune disease. Dysregulated levels and sustained secretion of proinflammatory cytokines by γδ and/or CD4 T cells have devastating effects on autoimmune disease progression. In a collagen-induced arthritis (CIA) model (resembling human rheumatoid arthritis), IL17-producing Vγ4/Vδ4^+^ T cells selectively increase in joints and lymph nodes. Depletion of γδ T cells by anti Vγ4 antibody, markedly reduced the disease severity score revealing its pathogenic nature (143). Interestingly, both Th17 and Tγδ17 are present in the joints but Th17 cells localize proximal to the bone, which facilitates its interaction with osteoclast. Selective depletion of Th17 cells abrogated the bone resorption suggesting that Th17 but not Tγδ17 cells are responsible for bone destruction. Thus, Tγδ17 cells may be responsible for enhancing joint inflammation and exacerbate CIA (144). In contrast, absence of Tγδ17 was reported in patients with rheumatoid arthritis and in murine model of autoimmune arthritis (SKG model) (145). The SKG mouse model has defects in the differentiation of Tγδ17 cells (94), which might result into low Tγδ17 cells in the inflamed joints. Thus, the role of Tγδ17 cells in autoimmune arthritis need to be evaluated comprehensively.

Tγδ17 also enhanced experimental autoimmune encephalo- myelitis (EAE) (mouse model for human multiple sclerosis). Upon immunization of mice with myelin oligodendrocyte glycoprotein (MOG) peptide in complete Freund’s adjuvant (CFA), Vγ4^+^CCR6^+^IL23^+^ γδT cells accumulate in the central nervous system (CNS), which expand by 20-fold in absolute number during development of clinical signs of the disease (72). In contrast, IFNγ-producing γδT cells are low in CNS and marginally increase during course of EAE (103). The mechanism behind aggravation of EAE could be attributed to restraining the development of Foxp3^+^ regulatory T cells (Tregs) functions by Tγδ17 cells. Supernatants from IL23-activated γδT cells inhibited the TGFβ driven conversion of naive Foxp3^−^ αβ T cells into Foxp3 expressing T cells and also reversed the suppressive effect of Treg cells (72). Similar function of Tγδ17 was reported in cardiac transplantation in mice. IL17, majorly produced by γδT cells, accelerates acute rejection of transplanted heart but IL17 deficiency enhanced Treg expansion and prolonged allograft survival (71). In ischemic brain injury, Tγδ17 were reported to be present at the infract areas (146). Tγδ17 rather than Th17 was the major source of IL17 whereas IFNγ was majorly produced by Th1 cells. In mice, genetically deficient for IL17 or IL23, the infract areas were reduced suggesting a role of Tγδ17 as a key contributor of neuroinflammation (146). Overall, this suggests that in chronic inflammatory condition, innate cytokines IL23 and IL1β promote infiltration and generation of IL17-producing γδT cells, which aggravate the disease.

Experimental silicosis is a useful model for depicting chronic lung inflammation, tissue damage, and fibrosis. Tγδ17 along with Th17 accumulated in the lung in response to IL23 expressing macrophages by third day after silica treatment but interestingly did not induce lung fibrosis (73). On the contrary, in allergic lung inflammation, Tγδ17 cells are known to be protective (147, 148). Functional blockage of both IL17 and γδT cells impaired the resolution of airway lung inflammation (148). It is claimed that this protective role is mediated by prostaglandins (PGs), which are abundant at the site of inflammation. PGI2 analog iloprost enhanced IL17 production by γδT cells in the thymus, spleen, and lungs, reducing airway inflammation (147). This highlights the role of PGI2 analogs that can be exploited in the development of immune response in immunotherapeutic approaches. Age-related macular degeneration (AMD) is another chronic inflammation associated disease, characterized by choroidal neovascularization (CNV). In an experimental model, Tγδ17 cells along with Thy-1^+^ ILCs (innate lymphoid cells) infiltrate the eye after laser treatment and promote neovascularization. This recruitment is in response to IL1β but not IL23 produced by macrophages (134).

## Tγδ17 Cells as Heroes or Villains in Cancer

The unmatched characteristics of human γδT cells to have MHC unrestricted tumor directed cytotoxicity, release of copious amounts of IFNγ, and recognition of cancer cells through variety of mechanisms render them as potential candidate for cancer immunotherapy (4, 149). Upon activation, γδT cells show cytotoxicity against myeloma (150), lymphoma (151), leukemia (152, 153), and other epithelial carcinomas (57, 154, 155) *in vitro*. Several clinical trials have been launched using γδT cells based therapies in cancer patients. The hallmark characteristic of γδT cells to be used for therapy is their ability to infiltrate tumors (156). *In vivo* activation by phosphoantigens or adaptive transfer of preactivated autologous γδT cells have proved successful in cancer treatment (157). However, the role of Tγδ17 cells as anticancer effector cells is not well defined.

In a chemotherapeutic approach, Tγδ17 cells are reported to play decisive role in several transplantable tumor models (EG7 thymoma, MCA205 sarcoma, CT26 colon cancer, and TS/A mammary carcinomas). Tγδ17 (Vγ4^+^/Vγ6^+^) cells were shown to invade the tumor bed early in response after drug treatment. This was followed by infiltration and induction of IFNγ-producing CD8 (Tc1) cells to the tumor bed. This infiltration of Tγδ17 and Tc1 cells was correlated and associated with tumor regression post radio or chemotherapy (158). Thus, IL17-producing Vγ4^+^/Vγ6^+^ cells are critical for the induction of Tc1 response in tumor tissue in response to drug treatment or radiation. Another study in bladder cancer supports the helper function of Tγδ17 cells in cancer treatment. Tγδ17 cells induce neutrophil infiltration to the tumor site and show anti-tumor effect upon *Mycobacterium bovis* BCG treatment (159).

In contrast to anti-tumor role of Tγδ17 cells, they also promote tumor development. With the notion that IL17 is a proangiogenic cytokine (160), Tγδ17 cells promote angiogenesis in tumor model. In IL17^−/−^ tumor bearing mice, the blood vessel density was markedly decreased compared to wild type. In addition, IL17 induced the expression of Ang-2 (angiopoietin) and VEGF (vascular endothelial growth factor) in tumor cells (8). In ovarian cancer model, it has been reported that CD27^−^ Vγ6^+^ cells produced higher IL17 and induce VEGF and Ang-2 in peritoneal exudates of tumor bearing mice after 6 weeks of post-tumor inoculation (161). Additionally, Tγδ17 cells induce mobilization of protumor small peritoneal macrophages (SPM) to the tumor bed, which express IL17-dependent proangiogenic profile (*Il1b, Il6, vegfa, tgfb, mif, cxcl1, cxcl8*, and *tie2*). SPMs also enhance ovarian cancer growth by stimulating tumor cell proliferation (161). In hepatocellular carcinoma mouse model, it was reported that IL17, majorly produced by Vγ4^+^γδT cells, induced CXCL5 production by tumor cells, which enhance migration of MDSCs (myeloid-derived suppressor cells) expressing CXCR2 to the tumor site. In addition, IL17 also enhanced suppressive functions of MDSCs by inhibition of T cells proliferation and cytokine (IFNγ and TNFα) production (162). In return, MDSCs induced γδT cells to produce IL17 through IL23 and IL1β secretion forming positive feedback loop for Tγδ17 activation (162). Thus, Tγδ17 cells interact with myeloid cells and counteract tumor immune-surveillance.

In human colorectal cancer, IL8 and GM-CSF secreted by Tγδ17 promote migration of MDSCs while IL17 and GM-CSF enhanced their proliferation. Tγδ17 cells also support survival of MDSCs through IL17, IL8, and TNFα (74). Thus, it is possible to speculate that Tγδ17 cells might be responsible for gradual shift from initial inflammatory to immunosuppressive tumor environment in advanced stage cancer (163). In human colorectal carcinoma, Tγδ17 cells were positively correlated with advancing tumor stages as well as with clinicopathological features including tumor size, tumor invasion, lymphatic and vascular invasion, lymph node metastasis, and serum CEA (Carcinoembryonic antigen) levels suggesting their pathogenic role (74).

Collectively, these findings highlight the apparently opposite roles of Tγδ17 cells in cancer immunity. It seems that during tumor development, inflammatory environment (IL1β and IL23) modulate the cytokine profile of γδT cells from primary IFNγ toward proinflammatory IL17, which support tumor progression.

## Concluding Remarks

**Figure 2:**
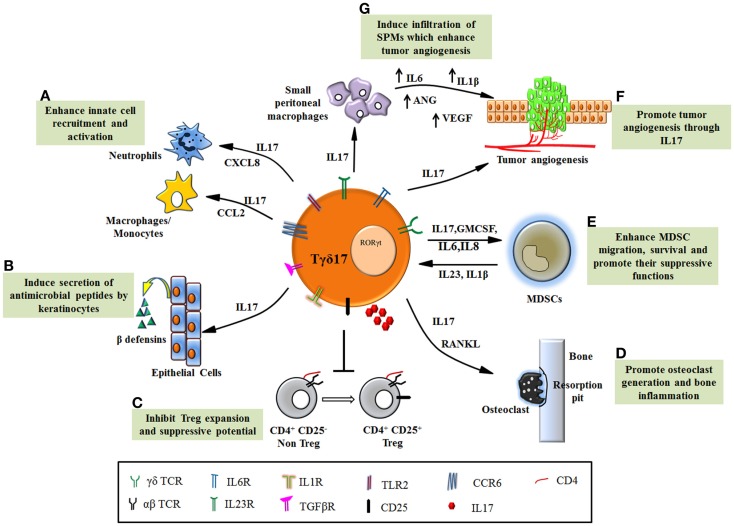
**Functions of Tγδ17 cells in pathological conditions**. **(A)** Tγδ17 cells promote infiltration of neutrophils and monocytes/macrophages to the site of inflammation through chemokines. **(B)** IL17 secreted by Tγδ17 cells induces keratinocytes to produce anti-microbial peptides such as β defensins and protect host in infections. **(C)** Dysregulated Tγδ17 cells in autoimmune diseases inhibit Treg expansion and its ability to suppress autoreactive cell, thereby exacerbating the disease. **(D)** The inflammatory condition in arthritis is worsened by IL17, which foster osteoclast formation through induction of RANKL. Tγδ17 cells are involved in bone resorption and enhance joint inflammation. **(E)** Human Tγδ17 cells support MDSC migration, survival, and promote their suppressive functions through IL17, GMCSF, and IL8. MDSCs also form feedback loop and promote Tγδ17 differentiation through IL23 and IL1β. **(F)** Tγδ17 cells secrete IL17 and induce tumorigenesis by their proangiogenic activity. **(G)** Murine Tγδ17 cells recruit small peritoneal macrophages to the tumor bed, which induce angiogenesis.

Despite the small percentage in total T cell population, γδT cells have emerged as an important modulator of early immune responses. The development of functional subtypes of γδT cells require polarizing cues including molecular and cellular interaction and combination of multiple cytokines and chemokine receptors that regulate their distribution. This suggests that the functional determination of γδT cell subtypes is dictated by the local environment (thymus or peripheral blood or the inflamed tissue) in which they are present. Tγδ17 is a special γδT cell subset, distinctly present at early immune response in the tissue and can modulate the functions of other immune and epithelial cells but their relevance in disease outcome remains controversial. In response to microbial antigens, Tγδ17 cells promote infiltration of neutrophils and macrophages and induce production of anti-microbial peptides resulting in clearance of microbial load. Such protective behavior of Tγδ17 cells in infections can be exploited to develop newer approaches to tackle the microbial pathology (Figure 2).

The opposite side of Tγδ17 functions has revealed its detrimental role in enhancing inflammation in autoimmunity and cancer (Figure 2). The mechanism, which regulates such dual personality of Tγδ17 cells is unknown. It appears that the obvious common role executed by these cells is enhancement of inflammation but due to functional heterogeneity and their complex interdependency on other cells (innate and adaptive); the emerging scenario of their biology is far from complete. This provokes us to consider contextual behavior of Tγδ17 cells in disease pathology. Current progress in understanding the significance of Tγδ17 cells in inflammatory diseases has revealed their novel but debilitating functions such as suppression of Tregs in autoimmunity, induction of angiogenesis, and recruitment and activation of MDSCs in various malignancies. Thus, in inflammatory disorders, Tγδ17 cells can be targeted using various immunotherapeutic approaches. However, need of hour is to expand the understandings of Tγδ17 in humans and develop a protocol for their propagation and activation. The future therapies will rely on regulating the key transcription factor RORγt by designing suitable antagonists that will help in fine tuning Tγδ17 differentiation and eventually their function in chronic inflammation and infection.

## Conflict of Interest Statement

The authors declare that the research was conducted in the absence of any commercial or financial relationships that could be construed as a potential conflict of interest.
